# Hip Abductor and Adductor Rate of Torque Development and Muscle Activation, but Not Muscle Size, Are Associated With Functional Performance

**DOI:** 10.3389/fphys.2021.744153

**Published:** 2021-10-14

**Authors:** Marcel Bahia Lanza, Kelly Rock, Victoria Marchese, Odessa Addison, Vicki L. Gray

**Affiliations:** ^1^Department of Physical Therapy and Rehabilitation Science, University of Maryland School of Medicine, Baltimore, MD, United States; ^2^Department of Veterans Affairs and Veterans Affairs Medical Center, Geriatric Research, Education and Clinical Centers, Baltimore, Maryland

**Keywords:** hip adductors, rate of torque development, maximal voluntary isometric contraction, electromyography, ultrasound, physical function, hip abductors

## Abstract

Understanding the physiological variables that contribute to a functional task provides important information for trainers and clinicians to improve functional performance. The hip abductors and adductors muscles appear to be important in determining the performance of some functional tasks; however, little is known about the relationship of the hip abductor/adductors muscle strength, activation, and size with functional performance. This study aimed to investigate the relationship of maximum torque, rate of torque development (RTD), rate of activation (RoA), and muscle thickness of the hip abductors [tensor fascia latae (TFL) and gluteus medius (GM)] and adductor magnus muscle with the Four Square Step Test (FSST) and the two-leg hop test in healthy young adults. Twenty participants (five males) attended one testing session that involved ultrasound image acquisition, maximal isometric voluntary contractions (hip abduction and hip adduction) while surface electromyography (EMG) was recorded, and two functional tests (FSST and two-leg side hop test). Bivariate correlations were performed between maximum voluntary torque (MVT), RTD at 50, 100, 200, and 300ms, RoA at 0–50, 0–100, 0–200, and 0–300, and muscle thickness with the dynamic stability tests. For the hip abduction, MVT (*r*=−0.455, *p*=0.044) and RTD_300_ (*r*=−0.494, *p*=0.027) was correlated with the FSST. GM RoA_50_ (*r*=−0.481, *p*=0.032) and RoA_100_ (*r*=−0.459, *p*=0.042) were significantly correlated with the two-leg side hop test. For the hip adduction, there was a significant correlation between the FSST and RTD_300_ (*r*=−0.500, *p*=0.025), while the two-leg side hop test was correlated with RTD_200_ (*r*=0.446, *p*=0.049) and RTD_300_ (*r*=0.594, *p*=0.006). Overall, the ability of the hip abductor and adductor muscles to produce torque quickly, GM rapid activation, and hip abductor MVT is important for better performance on the FSST and two-leg hop tests. However, muscle size appears not to influence the same tests.

## Introduction

Physical function, such as dynamic balance and agility, requires a shift of weight quickly to the supporting lower extremity to progress the body in any direction. During tasks that require weight shifting (e.g., lateral stepping), the hip abductors and adductor muscles appear to be critical for controlling this movement (Francis et al., 2018; Inacio et al., 2018; Lanza et al., 2020a). These muscle groups have also been found to be important in the control of weight bearing resistance training exercises (e.g., squats and lunges; McCurdy and Conner, 2003; Stastny et al., 2015). The hip abductor and adductor muscles may change with human development, given that physical function changes across the life span. With changes in physical function across age (Bohannon et al., 1984; Bohannon, 1997; Kolic et al., 2020), the control and proficiency of human movement that is determined by the ability of the muscles to produce torque, along with its underlying mechanisms (e.g., neuromuscular activation and muscle size), may also be affected. Thus, understanding how torque, muscle size, and activation contribute to different balance and agility tasks in young healthy adults is important to clinicians and trainers to target interventions to improve physical function and may provide normative data to compare between different age groups.

The ability to quickly produce torque is essential for better jumping (McErlain-Naylor et al., 2014) and sprinting performance (Maćkała et al., 2015). Additionally, quickly producing torque during the first moments of the contraction (up to the first 300ms from contraction onset) appears to be critical for successfully performing daily tasks such as stepping (Lanza et al., 2020b) and ascending stairs (Orssatto et al., 2020). Orssatto et al. (2020) found that the knee extensors rate of torque development (RTD, 0–200ms) explained 20% of the variance during an ascending stairs test in older adults, while other studies showed that hip abductors and adductors RTD (0–100, 0–150, and 0–200ms) explained up to 53% of the variance of the weight transfer preceding a step in older adults (Lanza et al., 2020a,b). Hence, if an individual can quickly produce torque, it is likely that physical function may be enhanced in many tasks.

Torque production is dependent on neuromuscular activation [measured from surface electromyography (EMG)] and muscle size (Folland et al., 2014; Maden-Wilkinson et al., 2021); hence, it plays an important role in physical function. A systematic review showed that higher neuromuscular activation of the agonist muscles measured during different tests (e.g., deep squat, star excursion balance test, among others) was critical for better performance (Kivlan and Martin, 2012). Also, rapidly activating the muscles (e.g., rate of activation, RoA) to stabilize the movement appears critical to physical function. For instance, higher RoA of the tensor fascia latae (TFL) and adductor magnus results in a shorter time to shift weight before initiating a step in older adults (Lanza et al., 2020b). Moreover, muscle size along with neuromuscular activation, also explain physical function. Larger muscles have constantly showed to influence sprint (Miller et al., 2020) and jump performance (Alegre et al., 2009). However, no relationship was found between muscle size and the weight shifting phase preceding a step (Lanza et al., 2020b). It is important to notice the weight shifting phase is a stabilizing activity, while the sprint and jumping are performance tasks, which may explain the differences in muscle size contribution among tasks. Considering muscle size may provide different contributions between tasks, further explore its role is warranted. This type of information is critical, as it provides valuable information about the physiological mechanisms that need to be targeted to improve physical function in different situations.

Physical function is measured using a range of different tests across the whole life span, including tests to measure dynamic stability and agility. For instance, dynamic stability is important for physical function and can be defined as successfully maintaining balance control during movement. To assess dynamic stability in different directions over an object (forward, sideways, and backward), the Four Square step test (FSST) is used in both older and young adults (Dite and Temple, 2002; Dawson et al., 2018; Mathurapongsakul and Siriphorn, 2018). In addition to that, agility tests are also often used to measure physical function. The two-leg side hop test is a speed and agility test part of the BOT-2 (Bruininks and Bruininks, 2005), that is widely used in children and young adults to assess functional performance (Phillips et al., 2020; Marchese et al., 2021). A distinct characteristic of many dynamic stability tests, including the FSST, and speed and agility tests, such as the two-leg hop test, is that a better score (e.g., lower time and/or higher count of jumps) indicates better physical function. Along these lines, it is possible that the ability to quickly produce torque and activate the muscles, as well as muscle size, would be important determinants of better performance on these tests. Thus, considering that it is possible for clinicians to manipulate the training variables to improve torque, neuromuscular activation, and muscle size (Diniz et al., 2020; Martins-Costa et al., 2021), further exploration of the underlying mechanisms of performing functional tests is important.

Therefore, the aim of this study was to investigate the relationship of maximum torque, RTD, RoA, and muscle size of the hip abductors (TFL and gluteus medius) and adductors muscles (adductor magnus) with the FSST and two-leg hop test in healthy adults. Based on previous research (Lanza et al., 2020a,b), we hypothesized that hip abductors and adductors maximum torque, RTD, and TFL, GM, and adductor magnus muscle RoA would be correlated with the dynamic stability and agility tests, while muscle size may not be associated with the functional performance in healthy young adults. Given the results from healthy adults are often used as a normative value to compare to other populations, for example, to understand how injury and disease may affect these mechanisms and dynamic stability performance, determining the relationships in young healthy adults is important.

## Materials and Methods

### Participants

Twenty participants (18–35years of age) volunteered for this study. Inclusion criteria: healthy young adults 18–35years old. Exclusion criteria were: (1) no engagement in sports at a higher than a recreational level; (2) no reported history of a neurological or muscular disorder; and (3) no lower extremity surgery. All participants were recruited from a single metropolitan region. The sample size for the bivariate correlation was calculated by the software GPower (version 3.1; Faul et al., 2007), and the following inputs were used: (a) Tails (two); (b) correlation p H1 (0.6, based on the average *R*^2^ from significant correlations from a previous study; Lanza et al., 2020b); (c) alpha (0.05); (d) Power (0.8); and (e) Correlation p HD (0). A sample size of 19 participants was required to achieve significance. This study was approved by the University of Maryland School of Medicine Institutional Review Board. Written consent was obtained for all participants.

### Overview

Participants attended one testing session within this cross-sectional study. All measures were conducted on the dominant limb. Limb dominance was determined by asking the participant which leg they use to kick a ball. The assessment session included ultrasound image acquisition, followed by familiarization with hip abduction and adduction tasks. Familiarization consisted of three submaximal contractions (30s rest between contractions), at 50% of the participant’s perceived rate for hip abduction and adduction. After familiarization, at least 3min rest was provided, and three maximal isometric voluntary contractions for hip abduction and hip adduction while surface EMG was recorded. After at least 3min of rest, the functional tests were performed as follows: FSST and the two-leg side hop test.

### Measures and Procedures

#### Maximal Voluntary Isometric Contraction

Hip abduction and adduction maximal torque were measured at the distal femur using a handheld dynamometer, Lafayette Manual Muscle Testing System model 01165 (Lafayette Instrument, Lafayette, IN, United States), with the participant positioned supine with the legs extended (0 degrees of knee flexion). The supine position provides a better control of the joint angle during the test. The dynamometer was placed approximately 3cm above the knee joint center on the lateral side of the thigh for hip abduction, and on the medial thigh for hip adduction. The participants were instructed to take a normal inhalation and slow expiration during maximal isometric voluntary contractions with the instructions, “hold the position you are placed in and kick as fast and hard as you can into the testing device.” Participants completed three maximal isometric voluntary contractions for 5s each, with a rest break for at least 30s, but no more than 2min in between trials. A rest of at least 2min was also given between tasks. Force data were transformed to torque by multiplying the length of the lever arm (distance between the hip joint axis and location of the dynamometer placement) by the force value in newtons (N). The maximum voluntary torque (MVT) was measured as peak torque during each of the three contractions and then was averaged for analysis. The handheld dynamometer recorded the signal at 40Hz, and the signal was exported to a computer using specific software (HHD/MMT download tool, Lafayette, IN, United States). RTD was measured across epochs of 50, 100, 200, and 300ms (RTD_50_, RTD_100_, RTD_200_, and RTD_300_, respectively) from torque onset to the respective time and was divided by the duration of the epoch (e.g., torque at 50ms/0.05s) as previously used (Lanza et al., 2020b). RTD was normalized by the peak torque (MVT) achieved during the same contraction, and an average value was calculated for the three contractions for hip abduction and hip abduction.

#### Neuromuscular Activation

**Figure 1:**
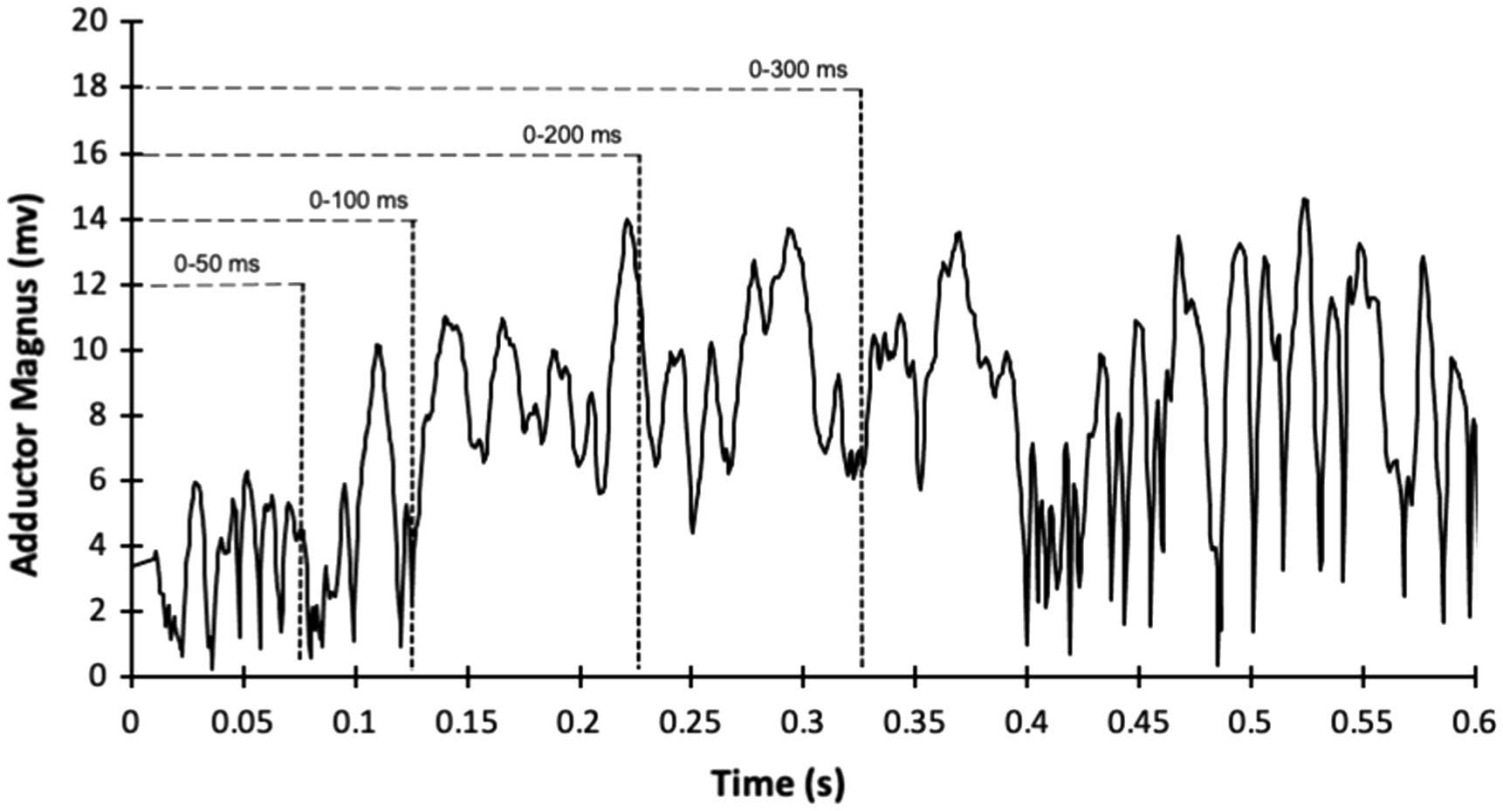
Example of sample recording of electromyography amplitude (filtered and root mean squared processed) during hip adduction maximum voluntary isometric contraction from the adductor magnus of one participant.

Surface EMG signals were collected using iWorx IX-BIO4 system (iWorx, Dover, NH, United States) during the hip abduction and adduction maximal isometric voluntary contractions. The electrodes were bipolar, disposable, pre-gelled, 1-cm-diameter, Ag/AgCl self-adhesive, circular snap electrodes with 20mm interelectrode spacing (Noraxon, Scottsdale, AZ, United States). The electrodes were placed on the muscle belly of the TFL and GM accordingly to the SENIAM guidelines (Hermens et al., 2000), while the adductor magnus electrode was placed at 1/3 of the superior line from the anterior superior iliac spine to the medial femoral epicondyle. A reference electrode was placed on the anterior tibia bone. The signals were bandpass filtered (6–500Hz bandwidth) and filtered using a fourth-order Butterworth digital bandpass filter. The EMG signal was separated into epochs, 0–50ms (RoA_50_), 0–100ms (RoA_100_), 0–200ms (RoA_200_), and 0–300ms (RoA_300_) from the EMG onset (Figure 1), as previously performed (Lanza et al., 2020b) and EMG signals were calculated as the root mean square across each epoch. After that, each average value was divided by the respective duration (e.g., EMG from 0 to 50 ms/0.05s) to generate the RoA. The EMG signal was normalized in each epoch by the highest EMG peak during the task (Besomi et al., 2020). The values were averaged for the three hip abduction and hip abduction contractions.

#### Ultrasound Image Acquisition

**Figure 2:**
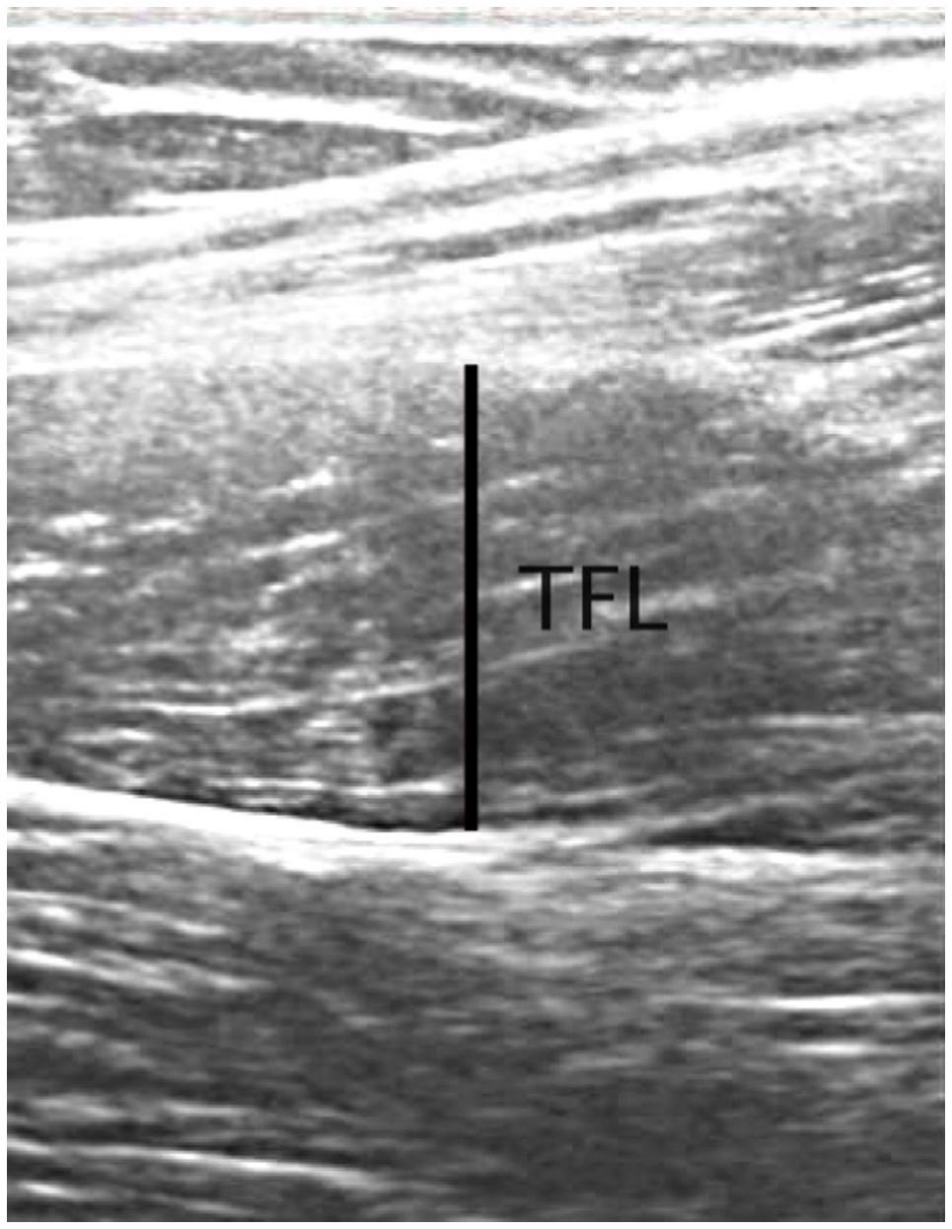
Example of ultrasonography image from the TFL muscle from one participant. Line indicates the location of muscle thickness measurements at 50% of the image.

Ultrasound images of the dominant limb were acquired from the TFL, and GM. Images were collected using 2D B-mode ultrasonography (Whale Sigma P5, Whale Imaging Inc., Waltham, MA, United States) with a 5–12MHz frequency, 38-mm linear array probe. All participants were positioned supine with the leg extended (0 degrees of knee flexion), and the hip in 0° abduction and rotation for the TFL, while for GM, participants were side lying with the dominant leg resting on the top of the non-dominant leg with the leg extended (0 degrees of knee flexion), and the hip in 0° rotation, and resting in approximately 20° of adduction (the resting, unsupported position of the test limb; Whittaker and Emery, 2014). The probe was placed parallel to the long axis of the muscle and perpendicular to the skin surface over the same location of the surface electrodes used during the strength testing. Ultrasound gel was used for good transmission, and the evaluator minimized any pressure from the probe onto the skin during the image acquisition. Images were imported into software (Tracker version 5.1.5; www.cabrillo.edu/~dbrown/tracker), and muscle thickness off-line analysis was conducted by one trained investigator (MBL). Three images for each muscle were collected for off-line analysis of muscle thickness (measured at 50% of the image size; Franchi et al., 2018; Figure 2), and the two images with good identification of the superficial and deeper aponeuroses, were used to analysis.

#### Physical Performance Tests

**Figure 3:**
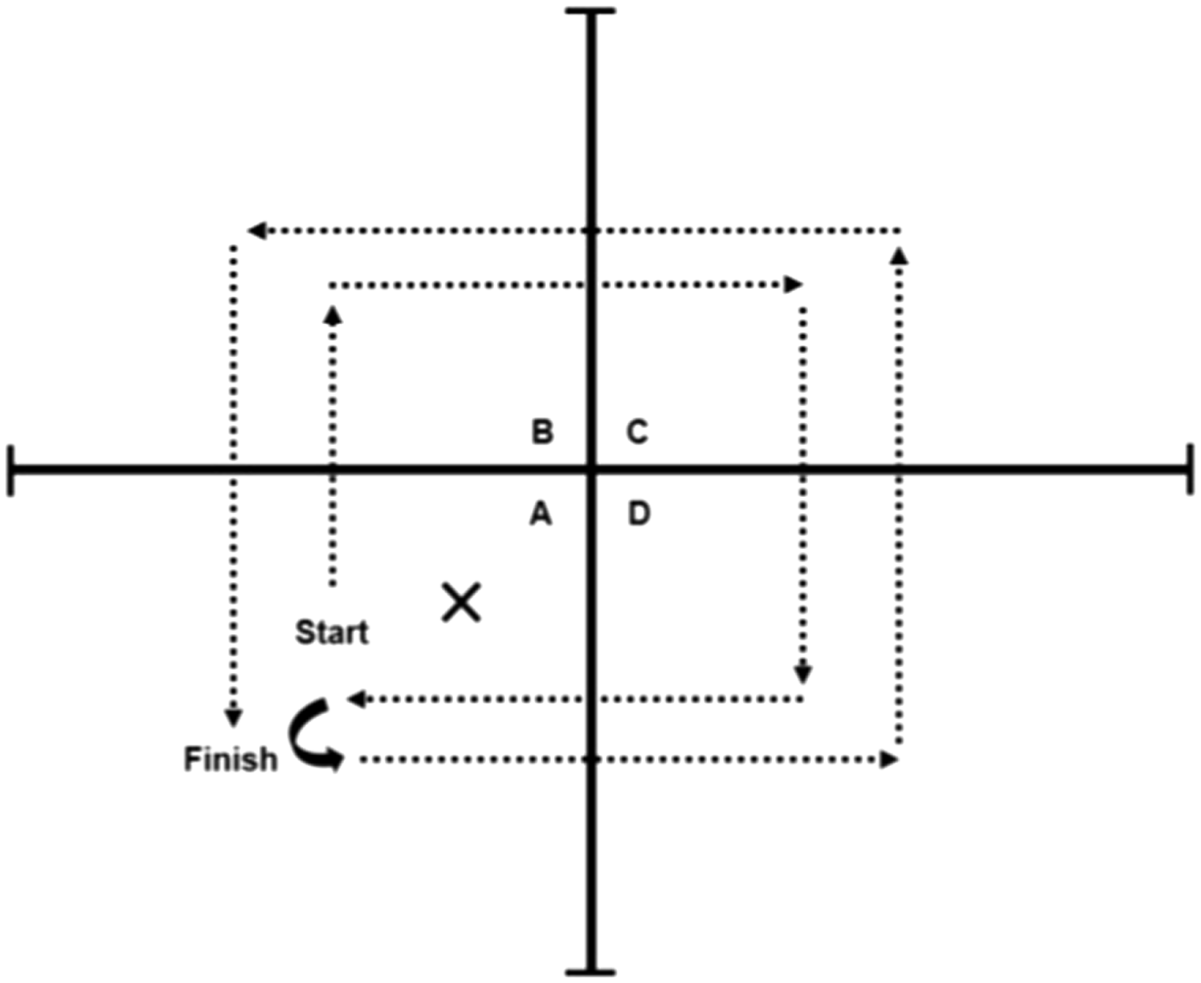
Four square step test schematics.

Participants performed the FSST, followed by the two-legged side hop test (Bruininks and Bruininks, 2005). During the FSST, the participant was instructed to step forward first and then continue stepping in each square in a clockwise sequence and then reverse the direction: B, C, D, A, D, C, B, and A (see Figure 3). All participants were instructed to complete the sequence as fast as possible without hitting the equipment (plastic tubes). Participants were given one practice trial, and then performed two test trials (Dawson et al., 2018). The best score (lowest time recorded) was used to perform the correlations. In the two-legged side hop test, participants were positioned next to and parallel to a line with hands-on-hips and feet together, and they were instructed to hop back and forth over the line until they were told to stop (number of jumps over 15s recorded). Both physical performance tests have excellent retest reliability with an ICC=0.98 for the FSST (Dite and Temple, 2002), and an ICC=0.87 for the two-legged side hop test (Bruininks and Bruininks, 2005).

### Statistical Analysis

A Shapiro-Wilk test was used to assess the normality of the data. Pearson’s product–moment bivariate correlations (r) were performed between RoA, muscle thickness, MVT, and RTD with the functional tests (FSST and two-leg side hop test), and forced entry multiple regression analysis was then performed with only the significant predictors from the correlation analyses entered into the model. Statistical analysis was performed using SPSS version 26 (IBM Corporation, Armonk, New York, United States); the significance level was set at *p*<0.05, data are presented as means±SD, and 95% CI are reported for correlation values.

## Results

Twenty volunteers participated in the study. The mean and SD of participants’ characteristics and functional tests are reported in Table 1, RTD and RoA are reported in Table 2, and muscle thickness measurements in Table 3.

**Table 1:** Demographics and dynamic stability tests of the study participants.

*n*	20
Males	5
Age (years)	28.7±4.9
Height (m)	1.70±0.11
Weight (Kg)	69.7±16.9
Dynamic stability tests	
Four Square Step Test (s)	5.6±0.9
Two-leg side hop test (count)	33.1±4.6

**Table 2:** Mean values and SDs of the hip abductor and adductor rate of torque development (RTD) and maximum voluntary torque (MVT), and the list muscles rate of activation (RoA).

Torque	
*Hip abduction*	
RTD50 (%MVT.s^-1^)	2.5±0.9
RTD100 (%MVT.s^-1^)	1.4±0.4
RTD200 (%MVT.s^-1^)	1.1±0.4
RTD300 (%MVT.s^-1^)	1.2±0.5
MVT (N.m)	112±23
*Hip adduction*	
RTD50 (%MVT.s^-1^)	4.1±1.1
RTD100 (%MVT.s^-1^)	2.2±0.5
RTD200 (%MVT.s^-1^)	1.5±0.3
RTD300 (%MVT.s^-1^)	1.4±0.4
MVT (N.m)	76±20
RoA	
*Tensor fascia latae*	
RoA50 (%MVT.s^-1^)	4.75±1.50
RoA100 (%MVT.s^-1^)	2.72±0.81
RoA200 (%MVT.s^-1^)	1.60±0.51
RoA300 (%MVT.s^-1^)	1.11±0.32
*Gluteus medius*	
RoA50 (%MVT.s^-1^)	3.37±1.32
RoA100 (%MVT.s^-1^)	2.01±0.81
RoA200 (%MVT.s^-1^)	1.35±0.56
RoA300 (%MVT.s^-1^)	0.99±0.38
*Adductor magnus*	
RoA50 (%MVT.s^-1^)	4.66±2.36
RoA100 (%MVT.s^-1^)	2.47±1.16
RoA200 (%MVT.s^-1^)	1.34±0.53
RoA300 (%MVT.s^-1^)	0.94±0.35

**Table 3:** Mean values and SDs of muscle thickness of the tensor fascia latae (TFL) and gluteus medius (GM) muscles.

*Muscle thickness (cm)*		FSST		Two-leg side hop test	
		*r*	*p*	*r*	*p*
Tensor fascia latae	2.9±0.6	0.042	0.861	−0.025	0.916
Gluteus medius	2.5±0.9	0.04	0.867	−0.024	0.921

Pearson correlations between muscle thickness of the TFL and gluteus medius with the Four Square Step Test (FSST) and the two-leg side hop test.

### Hip Abductors

**Figure 4:**
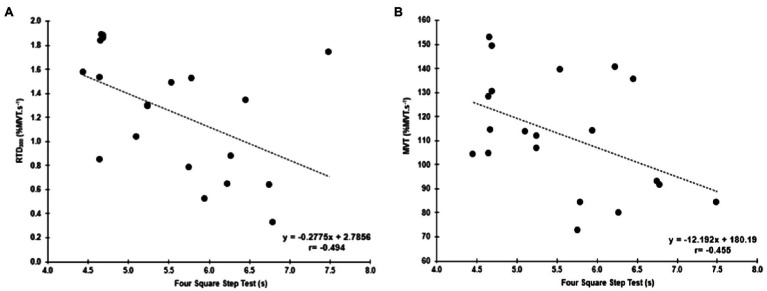
Correlations between the FSST and hip abductor normalized RTD at 300ms (RTD_300_; **A**) and hip abductor MVT **(B)**.

**Figure 5:**
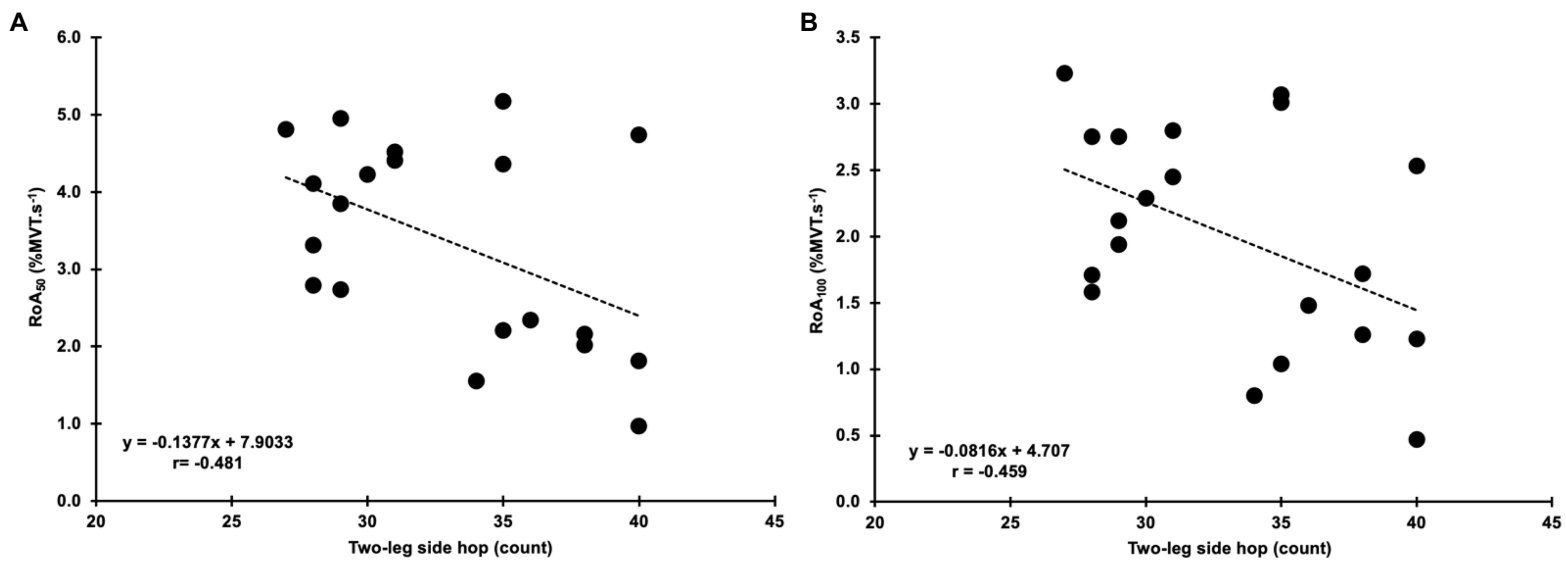
Correlations between the two-leg side hop test and gluteus medius normalized RoA at 50 and 100ms (RoA_50_ and RoA_100_; **A,B**, respectively).

Four Square Step Test was negatively correlated with MVT (*r*=−0.455, *p*=0.044, 95% CI: −0.733 to −0.098) and the RTD_300_ (*r*=−0.494, *p*=0.027, 95% CI: −0.886 to −0.024; Figure 4). Multiple regression analysis was calculated to predict FSST based on both (MVT and RTD_300_), and a significant regression equation was found [*F*(2,17)=4.305, *p*=0.031], with an *R*^2^ of 0.336. However, although the model was overall significant, both predictors were non-significant (MVT, *p*=0.142; RTD300, *p*=0.087). No significant correlations were found between FSST and the other hip abductor RTD time points (Supplementary Table A). No significant correlations were found between MVT and RTD and the two-leg side hop test (Supplementary Table A). GM RoA_50_ (*r*=−0.481, *p*=0.032, 95% CI: −0.808 to −0.035) and RoA_100_ (*r*=−0.459, *p*=0.042, 95% CI: −0.771 to −0.035) were significantly correlated with the two-leg side hop test (Figure 5), while no correlations were significant for the FSST. Multiple regression analysis was calculated to predict two-leg side hop based on both (GM RoA50 and GM RoA100), and the regression model was not significant. TFL RoA (Supplementary Table A) and muscle thickness (Table 3) were not significantly correlated with the functional tests.

### Hip Adductors

**Figure 6:**
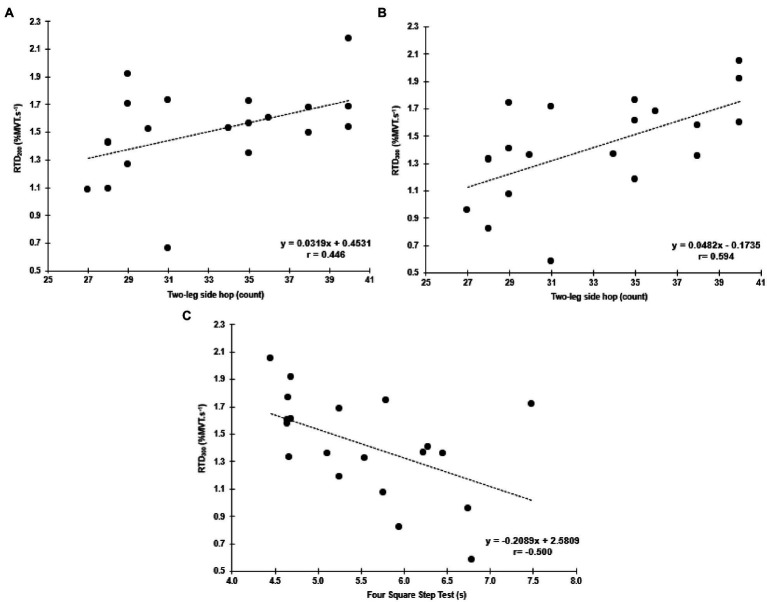
Correlations between the two-leg side hop test and hip adductor normalized RTD at 200 and 300ms (RTD_200_ and RTD_300_; **A**,**B**, respectively), and the FSST and RTD_300_ **(C)**.

No significant correlations were found with the functional tests and MVT. Similar to the hip abductors, there was a significant negative correlation between the FSST and RTD_300_ (*r*=−0.500, *p*=0.025, 95% CI: −0.845 to −0.046; Figure 6). However, contrary to the hip abductors, the RTD_200_ (*r*=0.446, *p*=0.049, 95%CI: 0.086–0.724) and RTD_300_ (*r*=0.594, *p*=0.006, 95% CI: 0.298–0.821) were significantly correlated with the two-leg side hop test (Figure 6). Multiple regression analysis was calculated to predict two-leg side hop based on both (RTD_200_ and RTD_300_), and a significant regression equation was found [*F*(2,17)=6.075, *p*=0.010], with an R2 of 0.417. In this model, only RTD300 was a significant predictor of the two-leg side hop test (*p*=0.022). Furthermore, no significant correlations were found between adductor magnus RoA and the FSST and two legged side hop tests (Supplementary Table A).

## Discussion

The aim of the present study was to investigate the relationship of MVT, RTD, RoA, and muscle thickness of hip abductors and adductors muscles with functional performance measured by the FSST and two-leg side hop test in young adults. We partially confirmed our hypotheses by demonstrating that overall, individuals with greater MVT and RTD during a maximal hip abduction and adduction contraction performed better during the FSST. However, only the ability of the hip adductor muscles to rapidly produce torque was associated with the two-leg side hop test. Neuromuscular activation in the early stages of the contraction (RoA_50_ and RoA_100_), only from the GM, appears to contribute to the two-leg hop test, while muscle thickness did not impact the functional performance, which also partially agreed with our hypotheses. Thus, overall, the ability of the hip abductor and adductor muscles to quickly produce torque as well as early GM neuromuscular activity appears to be important for functional performance (two-leg side hop). In contrast, muscle size (measured as muscle thickness) did not influence the functional performance of the tests used in this study.

The FSST requires lower limb movements in all directions (forward, backward, and lateral); hence, considering the hip muscles are working either as stabilizers, antagonists, or agonists of the movement, the contribution of these muscles to the functional performance tests was foreseen in our hypothesis. For the first time, we showed that the ability to rapidly produce hip abductor and adductors explosive torque (0–300ms) explained ~25% of the variance during the FSST. In comparison, hip abductors maximal torque explained ~21% of the variance during the FSST. Additionally, the ability of the hip adductors to quickly produce torque also explained ~20% (0–200ms) up to 35% (0–300ms) of the variance of the two-leg side hop test. The muscle results are relatively similar to previous research, which explored the earlier phases of torque production of the muscle groups (hip abductors and adductors) when older adults were performing a lateral voluntary step (Lanza et al., 2020a,b). Lanza et al. (2020a,b) reported that hip adduction MVT and RTD (up to 200ms) explained ~49% of the variance of the weight shifting phase of the choice reaction step test (Lanza et al., 2020b), while the hip abductors and adductor torque capacity explained up to ~55% of the variance of the reaction time during the same test (Lanza et al., 2020a). Moreover, it is very likely that other muscle groups (i.e., quadriceps muscles) influence the performance of these tests. Additionally, similar results were found in the plantar flexors where the ability to rapidly produce torque was associated with a single leg stance test (Ema et al., 2016). Ema et al. (2016) found that RTD (0–50 up to 0–200, with 50ms increments) explained up to 18% of the variance of the center of pressure displacement during the single-leg standing test (with eyes open) in older males (> 65years). However, when males and females were pooled together, RTD and MVT explained up to 6% of the variance of the single leg stance test, which is to some extent in line (but lower) with our findings (~20%) with young males and females pooled together but using different tests. Thus, the ability to quickly produce torque appears to be a critical factor underlying functional performance tests. It is important to note that the present study participants were highly functional, presenting lower values for the FSST, which may also influence our results. Also, future studies, with a larger sample size, should examine the differences between males and females RTD in relation to tests of dynamic stability to understand possible differences.

The muscle thickness was not associated with functional performance tests used in the present study. In corroborating our results, the hip abductors and adductors muscle size (measure as CSA) was not correlated with the weight transfer phase preceding a lateral voluntary step (Lanza et al., 2020b). These results reinforce that muscle thickness may not be as important as the ability to produce torque quickly during functional performance tasks. Although previous research showed that muscle size is one of the determinants of maximal (Maden-Wilkinson et al., 2020) and rapid torque production (Maden-Wilkinson et al., 2021), muscle size may not contribute to the same extent during submaximal tasks. Yet, we assessed muscle size by muscle thickness while some of the previous paper assessed by different techniques (e.g., resonance magnetic imaging, computerized tomography scan; Lanza et al., 2020b; Maden-Wilkinson et al., 2021), which may also explain possible differences between studies.

Additionally, we found no significant correlations for the TFL and adductor magnus, while GM RoA_50_ and RoA_100_ explained 23 and 21% of the variance during the two-leg side hop test, respectively. Our results reinforce the importance of some of the hip abductors muscle for functional performance. We previously showed that RoA of the TFL and adductor magnus (RoA at different time points) was strongly associated with the weight transfer phase preceding a lateral voluntary step (Lanza et al., 2020b). The difference in neuromuscular activation between studies might be due to the differences in duration and movement requirements of the tests. The two-leg side hop test and the FSST test are longer tests (15 and 5.6s, respectively, Table 1) than the weight shifting phase of the lateral voluntary step (~ 200ms). Additionally, while the weight transfer prepares the body for movement, the tests used here are more complex tasks that require several movements and also would require other muscles to be highly active during the task (e.g., quadriceps femoris muscle group). For instance, the FSST is multidirectional test that requires not only a step laterally, but also forward and backwards, while necessitating adequate foot clearance to avoid contact with the equipment. Thus, all these differences in test requirements may explain the difference in the importance of neuromuscular activation to functional tests between studies and population.

Considering only torque production, among the variables explored in this study, explained the variance of the functional performance tests used here; it is likely that other physiological mechanisms may also influence the test. The muscle contractile properties have been known to be one of the determinants of the ability to produce rapid torque (Lanza et al., 2019), and it has been related to fiber-type composition (Viitasalo and Komi, 1978) and muscle-tendon unit stiffness (Bojsen-Moller et al., 2005). Considering both tests we used required constant rapid movements, these factors are likely to also play a role in the current results. Moreover, considering how muscle structure (e.g., muscle size and architecture) changes across the life span (from children to older adults; Kubo et al., 2003), it is possible that the determinants of the functional performance tests may also change among different ages. Hence, further exploration to understand the changes across different ages and sexes would be important.

### Study Limitations and Relevance

In the present study, participants performed with relatively quick times on the FSST and high repetitions on the two-leg side hop test (Table 1). We also have a relatively small sample size (*n*=20) with more females than males; hence conclusions for bivariate correlations should be taken with caution. Another limitation is the use of a handheld dynamometer. Although the handheld dynamometer we used has shown to be valid and reliable (Martin et al., 2006; Rock et al., 2021), the torque measures may be underestimated compared to isokinetic dynamometers, which provide for a more rigorous test environment (Martin et al., 2006). Nonetheless, the handheld dynamometer is clinically applicable to use outside the laboratory; hence, our study provides an opportunity to be replicated in clinical settings. For example, if the assessment needs to be performed in a clinic, a hospital setting, or patient home the set-up used here might provide a great way to perform the testing given all the equipment can be easily carried with the evaluator (tape, stopwatch, and portable manual dynamometer).

Additionally, it is important to highlight how this information could be translated or used for other populations. The present study aimed to investigate healthy adults to expose the normative values within the physiological parameters and tests results for this population. Consequently, it is possible to understand the deviation that could occur in the underlying factors evaluated here, for instance, with aging, after an injury, or after a neurological condition (e.g., stroke) and use the present findings as a reference to prescribe exercise and improve physical function. Therefore, with caution, the functional tasks used in the present study, FSST and two-leg side hop test, could be applied in different populations (e.g., childhood to older adults) and would help assess children’s development or older adults’ functional performance. Furthermore, from a training perspective, to improve the ability to perform the FSST and the two-legged side hop test, increasing the ability to quickly produce torque at the hip abductors and adductors (respectively) appears critical.

In conclusion, the present study demonstrated that overall, the ability of the hip abductor and adductor muscles to produce torque quickly (RTD), the GM rapid activation (RoA_50_ and RoA_100_), and hip abductor MVT, are important for better performance on the FSST and two-leg hop tests. However, muscle size (measured as muscle thickness) appears to have no influence in the same functional tests. Further studies should explore these relationships in individuals after injury, surgery, and/or disease across the lifespan to aid in developing targeted treatment strategies.

## Data Availability Statement

The raw data supporting the conclusions of this article will be made available by the authors, without undue reservation.

## Ethics Statement

The studies involving human participants were reviewed and approved by University of Maryland School of Medicine Institutional Review Board. The patients/participants provided their written informed consent to participate in this study.

## Author Contributions

VG, VM, OA, KR, and ML conceived and designed the research. ML and KR performed data processing and analysis. All authors contributed to the article and approved the submitted version.

## Funding

OA was supported by a VA Career Development Award (IK2RX001788). ASR was supported by a VA Senior Research Career Scientist Award from Department of Veterans Affairs. ML was supported by a grant from the US Administration for Community Living, National Institute of Disability, Independent Living, and Rehabilitation Research post-doctoral training grant (90AR5028). KR was supported by a National Institute of Arthritis and Musculoskeletal and Skin Diseases (NIAMS) funded pre-doctoral fellowship (T32AR007592).

## Conflict of Interest

The authors declare that the research was conducted in the absence of any commercial or financial relationships that could be construed as a potential conflict of interest.

## Publisher’s Note

All claims expressed in this article are solely those of the authors and do not necessarily represent those of their affiliated organizations, or those of the publisher, the editors and the reviewers. Any product that may be evaluated in this article, or claim that may be made by its manufacturer, is not guaranteed or endorsed by the publisher.

## Abbreviations

CSA: Cross-sectional area
CT: Computerized tomography
EMG_MVT_: Electromyography amplitude from instantaneous maximal voluntary torque
GM: Gluteus medius
HDL: High-density lean tissue
IMAT: Intramuscular fat; MVT, maximal voluntary torque
RTD: rate of torque development
RoA: Rate of muscle activation
sEMG: Surface electromyography
