# Supplementary material for: Hip Abductor and Adductor Rate of Torque Development and Muscle Activation, but Not Muscle Size, Are Associated With Functional Performance
Source: Front Physiol. 2021 Oct 14;12:744153. doi: 10.3389/fphys.2021.744153 (PMC8551702; doi:10.3389/fphys.2021.744153)
Supplement: Supplementary file 1 [file Table_1.docx]

**Supplemental File**

**Table A**. Bivariate correlations between MVT, RTD at 50, 100, 200, 300 ms of the hip abductor and adductor muscles, and RoA at 50, 100, 200, 300 ms of the tensor fascia latae, gluteus medius and adductor magnus with the Four Square Step Test (FSST) and the two-leg side hop test.


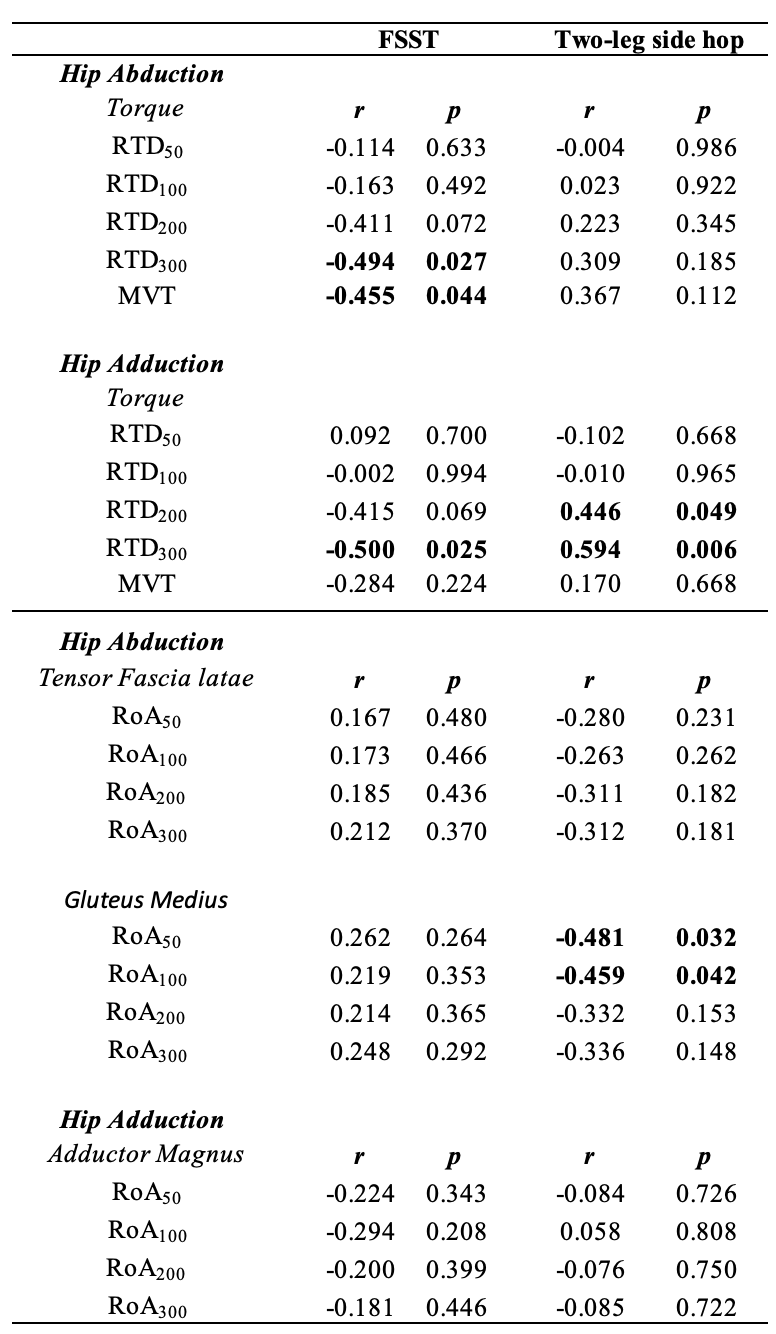


*

*

*

*

*

*

*

* Symbol indicates significant correlations.
